# Lipid metabolism in overweight/obese children vs. normal weight children in a north-eastern region of Spain

**DOI:** 10.1515/almed-2025-0015

**Published:** 2025-02-18

**Authors:** José Cuenca Alcocel, Lorena Villalba-Heredia, Inés Martínez Redondo, Alba Gallego Royo, José A. Casajús, José M. Arbonés-Mainar, Pilar Calmarza

**Affiliations:** Clinical Biochemistry Unit, Obispo Polanco Hospital, Teruel, Spain; GENUD Research Group, University of Zaragoza, Zaragoza, Spain; Pediatric Unit, Miguel Servet University Hospital, Zaragoza, Spain; Preventive Medicine Unit, Miguel Servet University Hospital, Zaragoza, Spain; GENUD (Growth, Exercise, Nutrition and Development) Research Group, University of Zaragoza, Aragon Health Research Institute (IIS Aragón), Zaragoza, Spain; Biomedical Research Center Network on the Pathophysiology of Obesity and Nutrition (CIBEROBN), Carlos III Health Research Institute, Madrid, Spain; Physical Medicine and Rehabilitation Unit, School of Health and Sports Sciences, University of Zaragoza, Zaragoza, Spain; Adipocyte and Fat Biology Laboratory (AdipoFat), Transversal Research Unit, Miguel Servet University Hospital, Aragon Health Research Institute (IIS), Zaragoza, Spain; Aragon Life Sciences Institute (IACS), Zaragoza, Spain; CIBER Obesity and Nutrition Pathophysiology (CIBERObn), Carlos III Health Institute, Madrid, Spain; Clinical Biochemistry Unit, Miguel Servet University Hospital, Zaragoza, Spain; Member of the SEQC^ML^ Research Groups on Oxidative Stress and Lipoproteins and Vascular Diseases, Research Project Center for Networked Biomedical Research on Cardiovascular Diseases (CIBERCV), University of Zaragoza, Aragon Health Research Institute (IIS Aragón), Zaragoza, Spain

**Keywords:** apolipoprotein A1, cholesterol, lipids, children, obesity, triglycerides

## Abstract

**Objectives:**

Obesity and overweight have increased in children and adolescents in Europe in the recent years, accounting for a major global public health problem. The objective of this study was the early detection of metabolic abnormalities in overweight/obese children (8–12 years old) that may ultimately induce impaired glucose metabolism and/or cardiovascular diseases.

**Methods:**

Lipid metabolism and metabolic control parameters were measured and monitored in a group of 61 male and female children with overweight/obesity and a group of 45 healthy, normal weight children, comparing the results obtained. Ages ranged from 8 to 12 years.

**Results:**

Higher levels of triglycerides and insulin and lower levels of high-density lipoprotein (HDL) cholesterol and apolipoprotein A1 were observed in overweight/obese children, as compared to normal weight children. Overweight/obese children exhibited higher apolipoprotein B/apolipoprotein A1 ratio, triglyceride-glucose ratio and HOMA index and a lower low-density lipoprotein (LDL) cholesterol/apolipoprotein B ratio.

**Conclusions:**

Obesity at an early age (8–12 years) negatively affects lipid parameters. Hence, overweight/obese children presented a more atherogenic lipid profile, manifested as higher concentrations of remnant particles and small dense LDL particles, higher insulin resistance and a higher risk for developing diabetes mellitus type 2 and cardiovascular disease, as compared to normal weight children.

## Introduction

Obesity and overweight in childhood and/or adolescence have increased in Europe in the recent years [1], accounting for a major global public health problem.

According to the International Obesity Task Force (IOTF) criteria [2], obesity and overweight are defined as a body mass index (BMI) exceeding the cut-offs established by Cole et al. for the equivalent of BMI 30 kg/m^2^ and 25 kg/m^2^, respectively.

Obesity is influenced by genetic and environmental factors [3]. Moreover, diet and physical activity play a major role in growth and body weight regulation. A poor diet added to sendentarism, among other factors, may result in child obesity and early atherosclerotic plaque formation. In this setting, subjects may develop even at a young age glucose metabolism disorder such as diabetes mellitus type 2 (DM2), lipid abnormalities or cardiovascular disease (CVD) [4]. Overweight/obesity contribute to the development of the so-called “metabolic syndrome” (MS) in adults and children [5]. MS may induce conditions such as non-alcoholic fatty liver disease, hypertension, coronary heart diseases and musculoskeletal problems. Additionally, overweight/obesity is a risk factor for the development of some types of cancer, poor school performance and mental disorders.

According to the 2019 ALADINO [6] and other studies [7], 8], the prevalence of child obesity/overweight in Spain has remained stable in the recent years; however, it is around 20 % in 6- to 12-year-old children. Interestingly, Spain is the second country with the highest prevalence of child overweight/obesity in Europe [9]. Considering the distribution of child overweight/obesity by sex, overweight is more prevalent among female children, whereas obesity has a higher prevalence among male children.

A variety of population-based studies [10] reveal that the prevalence of dyslipidemia in overweight and obese children and adolescents ranges from 20 % to more than 40 %, respectively. In contrast, only 8–20 % of normal weight children and adolescents have dyslipidemia [11].

The studies assessing dyslipidemia in obese/overweight children in our population are limited, and only a few examine metabolic and hormonal parameters. Of note, some of these studies unveiled an influence of obesity on the concentration of some hormones, including growth hormone, insulin [12], 13], leptin and intact parathyroid hormone (iPTH).

The purpose of this study was to determine a set of basic lipid parameters in obese/overweight children aged 8–12 years in a north-eastern region of Spain. The parameters studied included apolipoproteins A1 and B, lipoprotein (a) and metabolic parameters (insulin, insulin resistance). Results were compared to those obtained for age-matched normal weight children.

## Materials and methods

A comparative, observational study involving a group of overweight/obese and a group of normal weight children was performed in Zaragoza, Aragon, Spain. The overweight/obese group was composed of 61 male and female overweight/obese children, as established using the cut-offs defined by Cole et al. [2]. The cohort was extracted from the Exergames study, carried out at the University of Zaragoza and registered to clinicaltrials.gov (ID code NCT04418713). This study was approved by the Ethics Committee of the Regional Government of Aragon (Certificate No. 11/2018, CEICA, Zaragoza, Spain). The purpose of the Active Videogames (*Videojuegos Activos* after its original title) study, also known as “Exergames” [14], was conducted to examine the effects of an Active Videogaming and Multi-component Exercise program for prepubertal male and female children with overweight/obesity. The normal weight group was composed of 59 girls and boys with normal weight undergoing minor surgery (cryptorchidism, phimosis, trauma, among others). After a review of medical records, patients with confirmed disease and/or overweight/obesity established according to the International Obesity Task Force (IOTF) criteria [2] were excluded.

The final normal weight sample was composed of 45 children.

**Figure 1:**
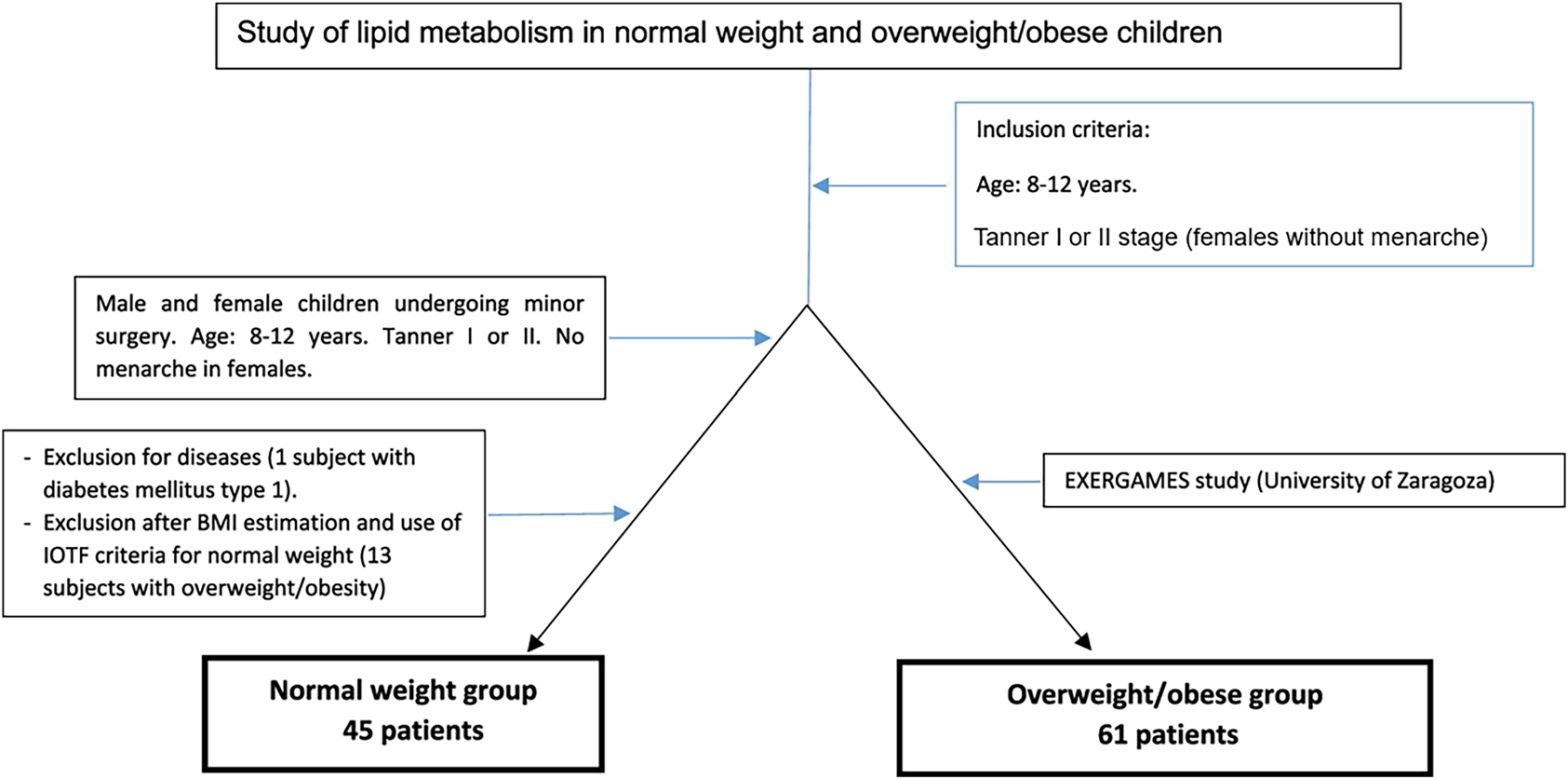
Selection of normal weight and overweight/obese children.

The two groups included 8 to 12 year-old children living in the autonomous community of Aragon who had not experienced the onset of puberty or menarche in girls (Tanner stages I and II). Children receiving some treatment or who had diseases that may influence study parameters (metabolic or chronic diseases, acute infection, anorexia nervosa) were excluded. Figure 1 displays the subject selection flowchart.

Parents received information about the study to be carry out and all signed the patient informed consent. Then, a short questionnaire was administered to collect epidemiological and clinical data. Anthropometric data included weight, height and body mass index (BMI). Physical examination and a fasting blood test were performed to determine the study parameters and evaluate lipid metabolism and metabolic profile in the study participants.

Blood samples were collected into serum-separator tubes, early in the morning after 8 h overnight fasting. The following basic lipid parameters were determined in serum: total cholesterol (TC), HDL cholesterol (HDL-C) and triglycerides (TG) in an AU 5800 Autoanalyzer (Beckmann Coulter Miami, FL, USA); LDL cholesterol (LDL-C) using the Friedewald formula; apolipoprotein A1 (Apo A1), apolipoprotein B (Apo B) and lipoprotein(a) (lp(a)) in an Immage Autoanalyzer (Beckmann Coulter Miami, FL, USA) by immunonephelometry; and Insulin in a Cobas e411 analyzer (Roche Diagnostics, Spain).

Additionally, Apo A1/Apo B, TG/HDL-C and LDL-C/Apo B ratios were calculated. The triglyceride-glucose index (TyG) was estimated using the following formula: Ln (triglycerides (mg/dL) × blood glucose (mg/dL)/2); the number of remnant particles was estimated using the formula: Total cholesterol – HDL-C – LDL-C; insulin resistance index (HOMA) using the formula: insulin (μU/mL) × blood glucose (mmol/L)/22.5; and quantitative insulin sensitivity check index (QUICKI) using the formula: 1/(log insulin (µU/mL) + log glucose (mg/dL)). Statistical analyses were performed using IBM SPSS Statistics version 26.0.

Firstly, the Lilliefors-corrected Kolmogorov-Smirnov (KSL) test was used to examine the distribution of quantitative anthropometric and biochemical variables. Parametric variables (KSL, p>0.05) were presented as means and standard deviation, whereas non-parametric variables (KSL, p*≤*0.05) were expressed as medians and interquartile ranges.

Normally-distributed anthropometric and biochemical variables were compared using Student’s t-test when variances in the two groups were homogeneous or Welck test when variances were non-homogeneous. Differences in non-parametric variables were examined using Mann Whitney U test.

A similar statistical analysis was performed using the tests described above to determine the suitability of the two study groups in terms of age, size and BMI, assessing their comparability in relation to these parameters.

To examine the influence of sex on overweight/obese and normal-weight children, each group was further divided into two sex-based subgroups. As a result, we obtained a subgroup of 34 overweight/obese male children and another subgroup of 27 overweight/obese female children. The normal-weight children group was divided into a subgroup of 29 male children and a subgroup of 16 female children. The tests mentioned above were used to assess significant sex-based differences between the two subgroups in the parameters and indices studied.

The overweight/obese group was also divided into a subgroup of overweight children and another subgroup of obese children by using the IOTF cut-offs. The resulting two subgroups included 40 obese and 21 overweight children, respectively. These subgroups were compared to assess significant differences between both subgroups in the parameters and indices studied.

The correlation between biochemical parameters and BMI, age, and sex was examined. The overweight/obese group was separated from the normal weight group to assess potential correlations with age and sex. The KSL test was first performed in the whole sample to assess the normality of variables. Pearson’s correlation coefficient was used when the variable followed a normal distribution, while Spearman’s correlation coefficient was applied when the distribution was non-normal. In the case of dichotomous variables (as in the case of sex), Kendall Tau-b test was performed. A p-value≤0.05 was established to identify statistical significance.

## Results

Table 1 contains the anthropometric data for the overweight/obese group and the normal weight group. No statistically significant differences were observed in terms of age between groups. The proportion of girls and boys was also similar in the two groups (55.7 % of boys in the overweight/obese group vs. 64.4 % in the normal weight group, p=0.367).

**Table 1:** Anthropometric data in normal weight and overweight/obese children.

	Overweight/obesity (n=61, 34 males and 27 females)^d^			Normal weight (n=45, 29 males and 16 females)^d^			Statistical significance (test)	Levene’s test p-value
	Total	Limits	Normality tests	Total	Limits	Normality test^c^		
Age, years	10.1 ± 0.9^a^	(8.4–12.2)	0.200	10.1 ± 1.1^a^	(8.4–12.0)	0.200	0.912 (Welch)	0.014
Weight, kg	55.4 (40.6–70.2)^b^	(33.4–89.1)	0.034	32.3 ± 5.4^a^	(22.0–42.0)	0.200	<0.001 (Mann-Whitney U test)	–
Height, cm	145 ± 8^a^	(129–161)	0.200	138 ± 9^a^	(119–155)	0.200	<0.001 (Student’s t-test)	0.521
BMI	25.9 ± 3.3^a^	(20.1–36.0)	0.200	17.1 ± 2.4^b^	(13.7–19.9)	0.023	<0.001 (Mann-Whitney U test)	–

	**Median**	**Limits**		**Median**	**Limits**			

Weight Z-score	2.55	(0.63–7.25)	–	−0.56	(−1.75–0.50)	–	–	–
Height Z-score	0.99	(−1.15–3.49)	–	−0.31	(−2.77–2.09)	–	–	–
BMI Z-score	2.80	(0.80–6.31)	–	−0.62	(−1.65–0.34)	–	–	–

^a^Mean±standard deviation; ^b^median (Q1-Q3); ^c^Lillefors-corrected Kolmogorov-Smirnov test; ^d^comparison of the sex distribution in the two groups by Pearson χ^2^ test *(*p=0.367).

Weight, height and BMI were statistically higher in the overweight/obese group (p<0.001), as compared to the normal weight group.

Laboratory results and statistical test results are shown in Tables 2 and 3.

**Table 2:** Distribution and comparative study of lipid parameters and insulin concentration of overweight/obese children vs. normal weight children.

Lipid parameters	Overweight/obese		Normal weight		Statistical significance (test)^d^	Levene’s test p-value
	Value	Normality tests^c^	Value	Normality tests^c^		
Total cholesterol, mg/dL	167.1 ± 32.6 (158.8–175.5)^a^	0.200	174.0 ± 24.6 (166.6–181.4)^a^	0.200	0.215 (Welch)	0.017
LDL cholesterol, mg/dL	98.7 ± 27.3 (91.8–105.7)^a^	0.194	100.2 ± 18.5 (94.6–105.7)^a^	0.073	0.747 (Welch)	0.003
Triglycerides, mg/dL	73.0 (61.5–98.5)^b^	<0.001	56.0 (45.5–69.9)^b^	0.004	**<0.001 (Mann-Whitney U test)**	–
HDL cholesterol, mg/dL	51.1 ± 9.6 (48.7–53.6)^a^	0.200	61.6 ± 13.8 (57.4–65.7)^a^	0.200	**<0.001 (Welch)**	0.012
Non-HDL cholesterol, mg/dL	116 ± 31 (107–124)^a^	0.171	112 ± 19 (107–118)^a^	0.175	0.473 (Welch)	0.001
Apo A1, mg/dL	148.1 ± 18.7 (143.3–152.9)^a^	0.182	172.6 ± 30.8 (163.3–181.8)^a^	0.200	**<0.001 (Welch)**	0.005
Apo B, mg/dL	81.5 ± 23.3 (75.5–87.4)^a^	0.200	76.6 ± 15.3 (72.0–81.2)^a^	0.180	0.195 (Welch)	0.002
Lipoprotein(a), mg/dL	14.5 (5.37–39.60)^b^	<0.001	10.7 (6.42–54.80)^b^	<0.001	0.924 (Mann-Whitney U test)	–
Insulin, μUI/mL	11.83 (8.95–17.15)^b^	<0.001	6.60 ± 2.71 (5.78–7.43)^a^	0.200	<0.001 (Mann-Whitney U test)	

^a^Mean±standard deviation (95%CI); ^b^median (Q1–Q3); ^c^Lillefors-corrected Kolmogorov-Smirnov; ^d^values in bold indicate statistically significant differences between groups for a 95 % confidence level. Apo A1, apolipoprotein A1; Apo B, apolipoprotein B.

**Table 3:** Distribution and comparison of lipid indices and insulin resistance in overweight/obese children vs. normal weight children.

Ratios and index	Overweight/obese children		Normal weight children		Statistical significance (test)^d^	Levene’s p-value
	Value	Normality tests^c^	Value	Normality tests^c^		
Apo B/Apo A1 ratio	0.56 ± 0.19 (0.51–0.61)^a^	0.099	0.46 ± 0.11 (0.43–0.49)^a^	0.200	**0.001 (Welch)**	0.008
TG/HDL cholesterol ratio	1.44 (1.14–2.05)^b^	<0.001	0.88 (1.41–0.66)^b^	0.001	**<0.001 (Mann-Whitney U test)**	–
LDL cholesterol/Apo B index	1.23 ± 0.18 (1.18–1.27)^a^	0.188	1.32 ± 0.14 (1.28–1.36)^a^	0.068	**0.004 (Student’s t-test)**	**0.215**
T&G indices	8.15 ± 0.45 (8.03–8.26)^a^	0.200	7.73 ± 0.11 (7.63–7.84)^a^	0.200	**<0.001 (Student’s t-test)**	**0.053**
HOMA index	2.64 (1.86–3.81)^b^	<0.001	1.40 ± 0.61 (1.20–1.61)^a^	0.200	**<0.001 (Mann-Whithey U test)**	–
QUICKI index	0.33 ± 0.03 (0.32–0.34)^a^	0.200	0.37 (0.35–0.39)^b^	0.035	**<0.001 (Mann-Whitney U test)**	–
Remnant particles, mg/dL	14 (12–19.5)^b^	<0.001	11 (9–14)^b^	<0.001	**<0.001 (Mann-Whitney U test)**	–

^a^Mean ± standard deviation (95%CI); ^b^median (Q1–Q3); ^c^Lillefors-corrected Kolmogorov-Smirnov test; ^d^values in bold indicate parameters with statistically significant differences between the two groups for a 95 % confidence level. TG, triglycerides; Apo A1, apolipoprotein A1; Apo B, apolipoprotein B; T&G, triglycerides-glucose.

The group of children with overweight/obesity showed significantly higher TG and significantly lower HDL-C concentrations, as compared to children with normal weight (p<0.001, in the two cases).

Remnant particle concentration was also statistically higher in the overweight/obese group (p<0.001).

The normal weight group exhibited higher levels of Apo A1, as compared to the overweight/obese group (p<0.001). In contrast, higher Apo B concentrations were observed in the overweight/obese group, although differences were not statistically significant (p=0.195). The LDL-C/Apo B ratio (p=0.004) was higher, whereas the TyG index and Apo B/Apo A1 ratio were lower in the normal weight group (p<0.001).

Statistically significant differences were also observed in insulin concentrations, which were higher in the overweight/obese group, as compared to the normal weight group. The HOMA and TyG indices were higher and the QUICKI index was lower in the overweight/obese group (p<0.001, in the two cases).

**Figure 2:**
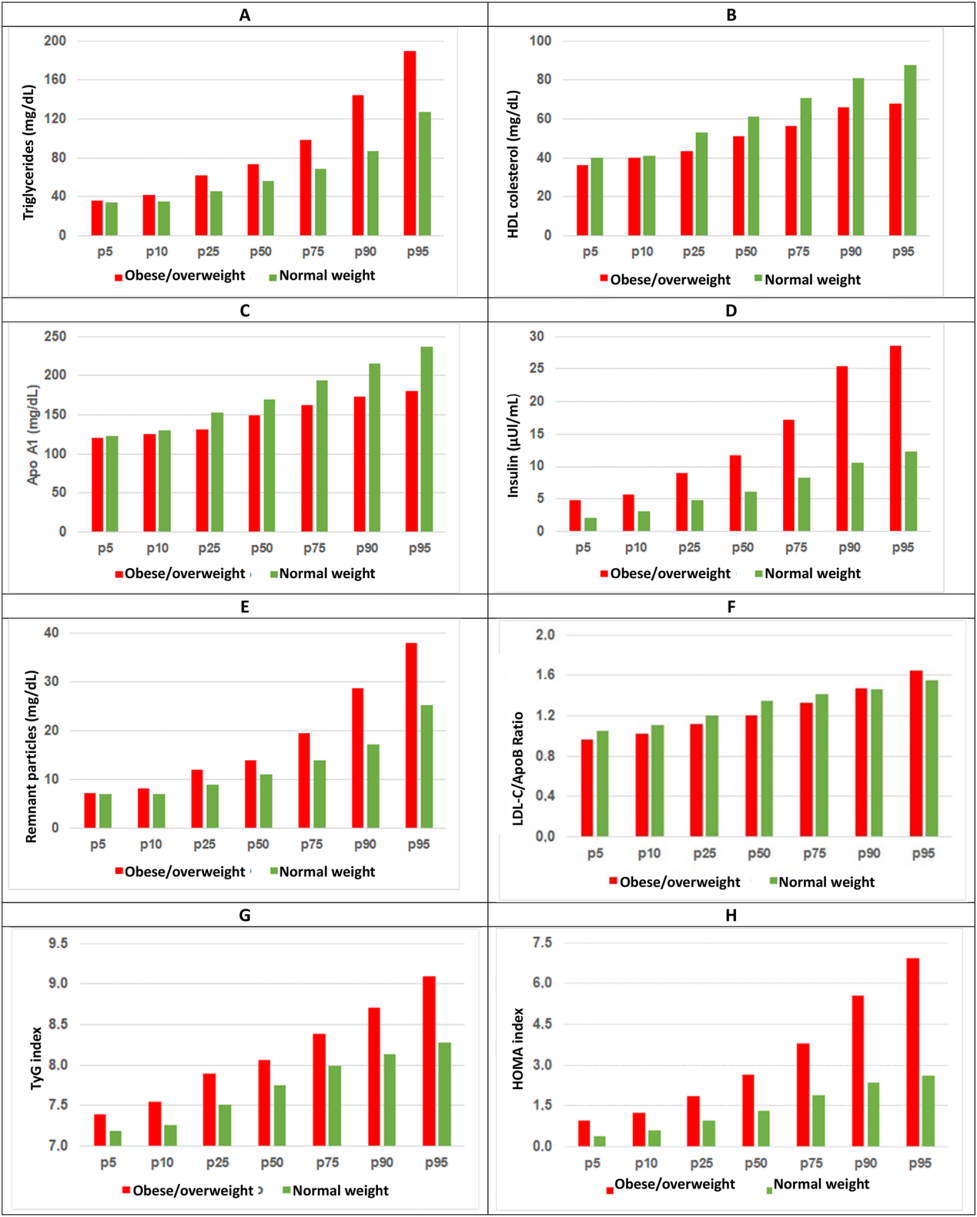
Percentiles for lipid and hormonal parameters in normal weight children and overweight/obese children. (A) Triglycerides, (B) HDL cholesterol, (C) Apo A1, (D) insulin, (E) remnant particles, (F) LDL-C/Apo B ratio, (G) TyG index, (H) HOMA index. LDL-C, LDL cholesterol; Apo A1, apolipoprotein A1; Apo B, apolipoprotein B; TyG, triglycerides-glucose.

Percentiles p5, p10, p25, p50, p75, p90 and p95 were calculated for the parameters, ratios and indices with statistically significant differences between the two study groups (Figure 2).

Considering the sex-based overweight/obese and normal weight subgroups, no statistically significant differences were found in any of the study parameters or ratios. In relation to normal weight subgroups, sex-based differences were only observed in the TyG index (males: 7.66* *±* *0.33; females: 7.88* *±* *0.27; p=0.041 (Student’s t-test)).

No significant differences were found between children with overweight subgroup and children with obesity subgroup in any of the parameters and ratios studied. A negative correlation was observed between BMI and Apo A1 (r=−0.460; p<0.001) and between BMI and HDL-C (r=−0.433; p<0.001). Additionally, BMI was positively correlated with TG (r=0.396; p<0.001) and insulin (r=0.683; p<0.001). In the normal weight group, there was a positive correlation between age and insulin (r=0.332; p=0.027) and between age and lipoprotein (a) (r=0.349; p=0.019). No significant correlation was observed between sex and the parameters studied.

A negative correlation was found between BMI and LDL-C/ApoB ratio (r=−0.238; p=0.014) and between BMI and QUICKI index (r=−0.677; p<0.001); BMI was positively correlated with ApoB/ApoA1 (r=0.277; p=0.004); TG/HDL-C (r=0.461; p<0.001); TyG (r=0.467; p<0.001); HOMA (r=0.677; p<0.001) and remnant particles (r=0.397; p<0.001).

Only in the normal weight group, age was positively correlated with HOMA (r=0.325; p=0.050) and TyG (r=0.450; p=0.005) and negatively correlated with the QUICKI index (r=−0.370; p=0.024). TyG (r=0.274; p=0.044) showed a positive correlation with female sex, only in normal weight group.

## Discussion

Overweight/obese children exhibited a clearly more atherogenic lipid profile than normal weight children. Thus, overweight/obese children have significantly higher levels of TG and lower levels of HDL-C and Apo A1, as compared to normal weight children. These results are consistent with previous studies [15], 16] reporting higher TG and lower HDL-C and Apo A1 concentrations in overweight/obese children. Nielsen et al. [17] documented higher levels of LDL-C, non-HDL-C and TC in male overweight/obese children, as compared to normal weight children. In contrast, no statistically significant differences were observed in these parameters in our study. Consistently with the study by Falaschett et al. [18], BMI was negatively correlated with Apo A1 and HDL. In addition, Apo B concentration was also higher in the overweight group, although differences did not reach significance in our study.

Apart from basic lipid parameters, triglyceride-rich lipoproteins and their metabolites play a major role in the residual risk of CVD. The reason is that these lipoproteins are highly atherogenic, due to their ability to penetrate and be retained in arterial walls, their high cholesterol content and their capacity to produce foam cells. These particles, known as remnant particles, which include chylomicrons, very-low density lipoproteins (VLDL) and intermediate-density lipoprotein (IDL) remnants are associated with the development of atherosclerotic cardiovascular disease [19], 20].

The group of overweight/obese children exhibited higher concentrations of remnant particles, as compared to normal weight group, which is consistent with the results obtained by Slyper et al. [21]. This finding suggests that overweight/obese faces an increased risk for developing atherosclerotic cardiovascular disease.

In relation to Apo A1 and Apo B concentrations, a meta-analysis conducted in 2020, including four papers and a total of 7,974 children and adolescents, revealed that the normal weight group had a 8.13 mg/dL higher mean Apo A1 concentration, compared with the 24.4 mg/dL higher mean Apo A1 concentration in the normal weight group, found in our study. In that study, significant differences were reported in Apo B concentrations between the two groups [22].

As mentioned above, TG/HDL-C and Apo B/Apo A1 ratios were higher in overweight/obese children, whereas the LDL-C/Apo B ratio was lower in the same group.

The Apo B/Apo A1 ratio is considered a major predictor of heart disease in adult obese patients [23]. Moreover, a number of studies in pediatric population report a positive correlation between this ratio and obesity, and an association of this ratio with a higher risk for cardiovascular disease [24], 25]. These results are in agreement with our study, which revealed that overweight/obese children presented a higher Apo B/Apo A1 ratio. Consequently, determination of this ratio can be useful for the early identification of obese children at a higher risk for developing CVD and atherosclerosis.

The LDL-C/Apo B ratio indirectly determines the number of small dense LDL, which are more atherogenic than other forms of LDL particles [26], 27]. A 1.2 ratio has been established to be equivalent to a size of 25.5 nm of LDL particles. It is also the cut-off for establishing a pattern of small dense LDL particles, provided when values of this quotient are equal to or below 1.2 [28]. The value of the LDL-C/Apo B ratio in predicting cardiovascular events with atherosclerotic disease [29] and its correlation with MS [30] has been widely demonstrated in adults. However, there is still insufficient data on its usefulness in the pediatric population. According to the results of our study, overweight/obese children have higher levels of small dense LDL particles and a more atherogenic pattern. Around 50 % of children in this group present a pattern of small dense LDL particles vs. around 25 % in the normal weight group. In a study in adult population, Xiao et al. [31] reported that patients who died from cardiovascular disease had a LDL-C/ApoB ratio <1.2, which was also lower than that of patients who did not die from cardiovascular events. Further studies are needed to assess the predictive value of this ratio in the pediatric population.

In relation to the TG/HDL-C ratio, there is evidence of a role in predicting insulin resistance (IR) and MS in overweight/obese children [32], [33], [34]. In our study, this ratio was found to be higher in overweight/obese children, which represents a higher risk for developing IR in this group. This ratio, along with the HOMA and QUICKI indices can be useful for the early identification of overweight/obese children with a higher IR and the associated higher risk for developing DM2.

Likewise, insulin, glucose concentrations and HOMA and TyG indices were higher in the overweight/obese group, whereas normal weight exhibited a higher QUICKI index.

The HOMA index is an estimation of IR from glucose and fasting insulin [35]. Higher HOMA values are associated with a higher IR [36]. Therefore, the overweight/obese children in our study had higher IR than normal weight children. Considering the cut-offs established for MS-associated IR, a meta-analysis conducted in 2019 [37] revealed that these cut-offs ranged from 2.30 to 3.54 for children and adolescents. According to the percentiles established for HOMA in our study, only 10 % of normal weight children would have IR vs. 50 % of overweight/obese children. In a similar study, Mastroeni et al. [38] also documented higher insulin concentrations and a higher HOMA index in overweight/obese children.

The QUICKI index can also be used to estimate IR. However, its correlation with the gold-standard method (hyperinsulinemic-euglycemic clamp) is slightly poorer than the one of the HOMA index [39]. In our study, overweight/obese children showed lower QUICKI values than normal weight children. These results are consistent with those obtained for the HOMA index, where overweight/obese children had higher IR. Sapunar et al. [40] also reported higher HOMA and lower QUICKI values in overweight/obese children, as compared to normal weight children.

The overweight/obese group also showed higher TyG indices. Like HOMA and QUICKI, the TyG index correlates with the development of IR [41], thereby having a role in assessing the risk for MS [42]. Different TyG cut-offs have been established for diverse populations to predict insulin resistance, based on the HOMA index. Dikaiakau et al. established a cut-off of 7.91 for predicting IR. Based on this cut-off, near 75 % of overweight/obese children in our study could have IR vs. 25 % of normal weight children. In our study, we have also found that children with a higher BMI had a more atherogenic profile and higher IR than normal weight children. Regarding age and sex, normal weight children had slightly higher HOMA and TyG index and a slightly lower QUICKI index as they grew older. The correlation of these parameters with BMI is more intense than with age. A slightly significant correlation was also found between the TyG index and sex, with female children showing slightly higher values than males. These results are in agreement with previous studies revealing an increase in the HOMA index and a decrease in the QUICKI index as children grow [35], 43].

The small sample size is a limitation of this study. However, this study is based on a well-characterized cohort of children at prepubertal age, which is a rarely studied population. This study could be extended to separate overweight from obese children, assess the influence of age and sex, and investigate other more sensitive lipid parameters to establish metabolic and cardiovascular risk in these children.

In conclusion, the group of overweight/obese children with ages from 8 to 12 years exhibited a more atherogenic lipid profile, higher concentrations of remnant and small dense LDL particles – being all very atherogenic – and higher insulin resistance, as compared to normal weight children. This difference involves a higher risk for overweight/obese children to develop DM2 and CVD.
